# Mechanisms of Maximum Information Preservation in the *Drosophila* Antennal Lobe

**DOI:** 10.1371/journal.pone.0010644

**Published:** 2010-05-21

**Authors:** Ryota Satoh, Masafumi Oizumi, Hokto Kazama, Masato Okada

**Affiliations:** 1 The University of Tokyo, Kashiwa-shi, Chiba, Japan; 2 Department of Neurobiology, Harvard Medical School, Boston, Massachusetts, United States of America; 3 RIKEN Brain Science Institute, Wako-shi, Saitama, Japan; Center for Genomic Regulation, Spain

## Abstract

We examined the presence of maximum information preservation, which may be a fundamental principle of information transmission in all sensory modalities, in the *Drosophila* antennal lobe using an experimentally grounded network model and physiological data. Recent studies have shown a nonlinear firing rate transformation between olfactory receptor neurons (ORNs) and second-order projection neurons (PNs). As a result, PNs can use their dynamic range more uniformly than ORNs in response to a diverse set of odors. Although this firing rate transformation is thought to assist the decoder in discriminating between odors, there are no comprehensive, quantitatively supported studies examining this notion. Therefore, we quantitatively investigated the efficiency of this firing rate transformation from the viewpoint of information preservation by computing the mutual information between odor stimuli and PN responses in our network model. In the *Drosophila* olfactory system, all ORNs and PNs are divided into unique functional processing units called glomeruli. The nonlinear transformation between ORNs and PNs is formed by intraglomerular transformation and interglomerular interaction through local neurons (LNs). By exploring possible nonlinear transformations produced by these two factors in our network model, we found that mutual information is maximized when a weak ORN input is preferentially amplified within a glomerulus and the net LN input to each glomerulus is inhibitory. It is noteworthy that this is the very combination observed experimentally. Furthermore, the shape of the resultant nonlinear transformation is similar to that observed experimentally. These results imply that information related to odor stimuli is almost maximally preserved in the *Drosophila* olfactory circuit. We also discuss how intraglomerular transformation and interglomerular inhibition combine to maximize mutual information.

## Introduction

How is sensory information received by sensory receptor cells transferred to higher brain regions? The data processing inequality of information theory states that any kind of information processing can only reduce the amount of information [1]. Sensory information is therefore gradually lost as it is passed to the next processing stage. However, for sensory information to be conveyed accurately to higher brain regions, as much information as possible should be preserved. Thus, it is conceivable that a principle common to all sensory modalities is ‘to maximally preserve the information’ [2]. Here, we investigated the presence and mechanisms of maximum information preservation in the olfactory system using a network model and physiological data of neural responses [3], [4].

**Figure 1:**
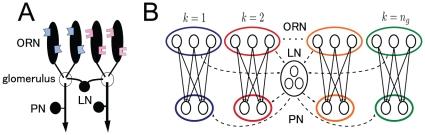
Schematics of the *Drosophila* olfactory circuit. (A) *Drosophila* olfactory circuit. (B) Circuit network model.

We chose the *Drosophila* antennal lobe as a model circuit because it has many advantages for investigating information transformation within the circuit. First, it is organized into discrete compartments termed glomeruli as in the vertebrate olfactory bulb (Fig. 1 (A)) [5], [6]. All olfactory receptor neurons (ORNs) expressing the same odorant receptor gene send their axons to the same glomerulus, where they synapse onto second-order projection neurons (PNs) [7], [8]. The dendrite of each PN is confined within a single glomerulus [9]–[11]. Local neurons (LNs) interconnect glomeruli and mediate both excitation and inhibition [6], [12]–[22]. This glomerular architecture simplifies physiological investigations of the circuit's connectivity. Second, there are only approximately 50 glomeruli in *Drosophila* [5] compared with approximately 1800 in mice. In each glomerulus, about 40 ORNs converge onto an average of three PNs [23]–[26]. Third, the responses of ORNs and PNs to various odors have been extensively analyzed [3], [4], [23], [24], [27]. These advantages enabled us to study information processing in the olfactory system on the basis of an olfactory network model that takes account of (1) the actual connectivity, (2) almost all neurons engaged in the olfactory processing, and (3) the response properties of ORNs and PNs to real odorants.

Importantly, because odor information in both the ORN and PN layers is represented by population activities of various types of ORNs and PNs [3], [4], [27], [28], the investigation of information processing in the olfactory system requires consideration of as many of the neurons that contribute to information processing as possible. However, quantitative assessment of information processing in large neuronal populations is difficult and few studies have examined large neural populations engaged in sensory information processing [29]. Here, we utilized the above three advantages to construct a network model that includes approximately half of all the neurons engaged in olfactory information processing and computed the amount of information contained in the entire neural population.

Recent investigations have shown that PNs are broadly tuned to odors, whereas ORNs are narrowly tuned [3], [30]. In ORNs, most odor responses cluster in the weak end of their dynamic range. In PNs, however, odor responses are distributed more uniformly throughout their dynamic range. This is a result of nonlinear transformation between ORN and PN responses. The nonlinearity amplifies weak ORN inputs greatly, but does not amplify strong ones as much. As PNs use their dynamic range more efficiently than ORNs, this transformation is thought to assist the decoder in discriminating between different odors. However, it is also expected that the neural variability of PN responses will increase when weak ORN inputs are amplified strongly. Confirmation that nonlinear transformation does increase odor discriminability requires quantitative verification that considers not only the separation of mean neural responses, but also the variability of responses. In this study, we quantitatively determined whether the nonlinear firing rate transformation was optimum in terms of maximum information preservation by computing the mutual information between odorant stimuli and PN responses in our network model. Mutual information quantifies odor discriminability taking into account not only the separation of mean neural responses but also the variability of responses without any assumption of specific decoders.

In the *Drosophila* antennal lobe, two main processes contribute to transform neural representations in ORNs into those in PNs, namely intraglomerular transformation and interglomerular interaction through LNs. The shape of the nonlinear transformation between ORN and PN firing rates is therefore formed by these two factors [3], [12]–[16], [31]. We simply parameterized the form of intraglomerular transformation as one variable and the strength of LN input to each glomerulus as another variable. By systematically varying these two variables, we found that mutual information between odor stimuli and PN responses was maximized when the intraglomerular transformation preferentially amplified a weak ORN input and the net LN input was inhibitory. This is the very combination observed experimentally [13], [31]. Furthermore, the shape of the resultant nonlinear transformation was similar to that obtained experimentally [3]. These results suggest that ORN activity is transformed into PN activity in a near-optimal manner so as to preserve the maximum information. We also discuss how the intraglomerular transformation and interglomerular interaction contribute to increase mutual information.

## Methods

### Network model of the *Drosophila* antennal lobe

In this section, we describe the construction of a network model of the *Drosophila* antennal lobe (Fig. 1 (B)). There are three types of neurons in the *Drosophila* antennal lobe: ORNs, PNs, and LNs. We assume that these neurons fire according to a Poisson process with a time-independent firing rate for ORNs and a time-dependent firing rate for PNs and LNs. Our network model has a two-layer feed-forward architecture consisting of an ORN layer and a PN layer. The antennal lobe is subdivided into characteristic structures called glomeruli that constitute discrete processing channels. All the ORNs expressing a particular receptor converge onto the same glomerulus and connect to PNs [7], [8], with the dendritic arbors of individual PNs confined within a single glomerulus [9]–[11]. Each PN therefore receives direct input from just one ORN type (Figs. 1 (A) and (B)). Our model incorporates all these characteristics of the antennal lobe circuit.

**Figure 2:**
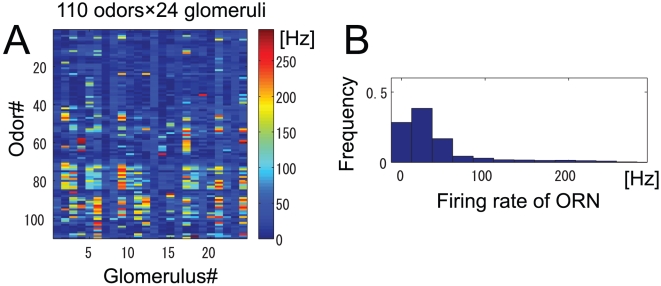
Properties of ORN responses. (A) Experimental data for 24 types of ORN responses to 110 odors adapted from Fig. 1 in Hallem & Carlson [4]. Colors show ORN firing rates. (B) Histogram of ORN response magnitudes obtained from the data in panel (A).

First, for the model of ORNs, we assumed that ORNs show only excitatory responses to odors and that these responses are time-independent. We determined the ORN firing rates for a given odor 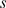 by using Hallem and Carlson's [4] comprehensive study, which measured responses of 24 types of ORNs to over 100 odors. The value of the mean ORN firing rate in response to odor 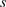 is denoted by 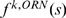, where superscript 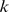 indicates the glomerular identity. The values of 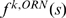 are shown in Fig. 2 (A) (Fig. 1 in ref. [4]). Figure 2 (B) shows a histogram of the ORN firing rate. Most ORN odor responses are clustered at the weak end of the dynamic range of the ORNs, with this being a characteristic feature of their responses.

Second, for the LN model, we assumed that (1) LNs receive synaptic input from all ORNs and (2) LNs innervate all PNs. These assumptions were made in order to reflect recent experimental observations that the strength of an inhibitory lateral input was positively correlated with the total ORN activity evoked by each odor [13] and that all the PNs examined received interglomerular excitation [12]. The former observation would imply that the odor tuning of the lateral input is similar across glomeruli. For simplicity, we assumed that the synaptic strengths between ORNs and LNs and between LNs and PNs are homogeneous. The total inputs from ORNs to LNs are modeled by
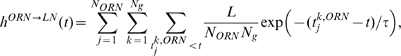
(1)where 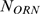 is the number of ORNs within a single glomerulus, 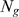 is the total number of glomeruli in the network, 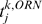 is the time when the 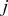
th ORN in the 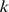
th glomerulus fires, and 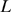 is the synaptic strength between ORNs and LNs. 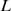 is set to 10. Synaptic inputs from ORNs are modeled by an exponential with a time constant 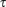. We set 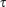 to 2 ms. The results of our study were insensitive to the absolute value of 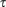 (data not shown). Synaptic events before time 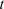 are all summed up linearly in Eq. 1. We assumed that the LN firing rate 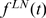 increases linearly with the strength of input 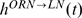, i.e.,
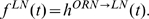
(2)


**Figure 3:**
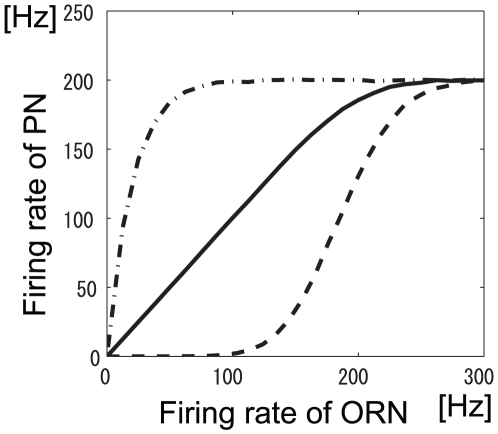
Transformation between ORN and PN firing rates for various values of 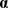. LN input was set to 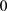 (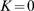). The PN firing threshold, 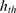, is 0. 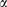 is 0 (solid line), 30 (dashed line), −30 (dot-dashed line).

Third, for the PN model, we chose a configuration in which each PN receives direct input from ORNs in a single glomerulus, 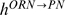, and lateral input from LNs, 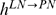. Therefore, the total inputs received by a PN at time 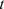 are modeled by
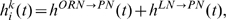
(3)

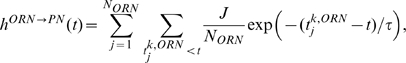
(4)

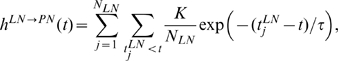
(5)where 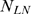 is the number of LNs and 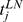 is the time when the 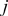
th LN fires. In Eq. 4, 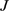 is the synaptic strength between ORNs and PNs. We chose a configuration where ORNs are connected to PNs in an all-to-all manner and the synaptic strength between ORNs and PNs is homogeneous, reflecting the experimental findings [31], [32]. In Eq. 5, 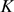 is a parameter controlling the strength of lateral input from LNs. Lateral input is excitatory when 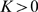 and inhibitory when 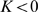. Although the net LN input is inhibitory, as observed experimentally [13], excitatory LNs are also present within the antennal lobe [12], [14], [15]. We examined the effects of both excitatory (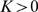) and inhibitory (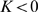) lateral inputs on odor discriminability on the basis of PN responses. The PN firing rate at time 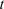 is determined by the strength of input 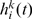 as
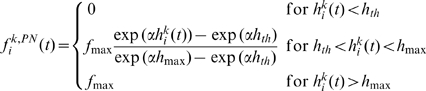
(6)where 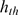 is an input threshold value below which the PN firing rate is 0 and 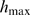 is the value above which the PN firing rate is saturated at the maximum value 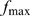. The relationship between ORN and PN firing rates for different 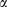 values when 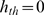, 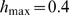, 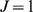, and 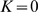 (no lateral input) is shown in Fig. 3. Here, 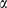 controls the shape of the transformation between ORN and PN firing rates within a glomerulus. The functional form of Eq. 6 suitably describes the actual relationship between ORN and PN firing rates [3], [13]. When 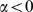, the intraglomerular transformation preferentially amplifies weak ORN inputs and when 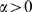, it rather suppresses weak ORN inputs. We call the firing rate transformation concave when 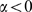 and convex when 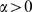.

From Eqs. 3–6, we can see that the strength of feed-forward connections between ORNs and PNs 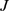 is just a scaling parameter, i.e., free parameters are only the ratio of strength of feed-forward and lateral connections, 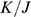, and 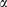. For simplicity, we set 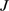 to 1 without loss of generality. Parameters 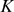 and 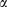 determine the relationship between ORN and PN firing rates. We investigated the optimum firing rate transformation between ORNs and PNs from the viewpoint of maximum mutual information by systematically changing 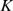 and 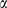.

**Figure 4:**
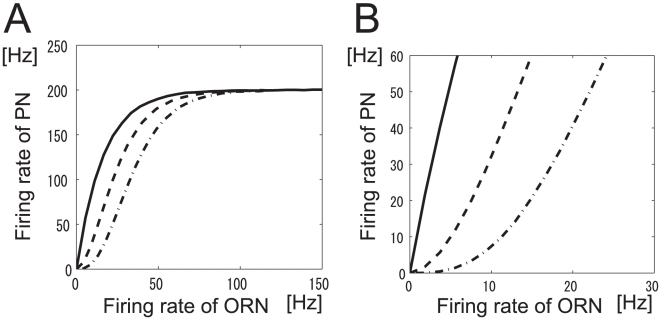
Transformation between ORN and PN firing rates for various values of firing threshold. LN inputs were set to 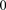 (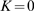). 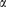 was set to 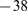. The PN firing threshold, 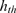, is 0 (solid line), 0.02 (dashed line), or 0.04 (dot-dashed line). Panel B is an expanded view of panel A.

The parameters 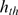 and 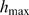 were fixed as follows. First, we determined 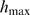 so that the PN firing rate saturates when the firing rate of a presynaptic ORN is nearly 250 Hz (Fig. 3). Specifically, 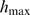 was set to 0.4. Second, we determined 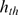 based on the experimentally observed relationship between ORN and PN firing rates without lateral input [13]. This experiment showed that the slope of the ORN-to-PN firing rate transformation close to the origin was very steep in the absence of lateral input. This indicates that 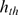 is very small. The relationship between ORN and PN firing rates for different values of 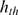 is shown in Fig. 4. When 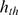 is 0.04 (dot-dashed line in Fig. 4), the slope at the origin is nearly 0. This is inconsistent with the experimental data [13]. For simplicity, we set 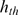 to 0 (see ‘Effect of static firing threshold on mutual information’ for cases of different 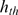). When we determine 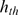 and 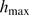 as described above, our model emulates the experimentally observed firing rate transformation in [13] for a certain value of 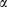.

### Mutual information

We computed the mutual information between population activities of PNs 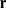 and odors 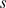. A component of vector 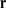 is the number of spikes emitted by a PN within a time bin 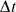. We set 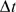 to 10 ms. Since we defined the maximum PN firing rate as 200 Hz, two spikes are emitted on average by PNs with the highest firing rate. To reduce the amount of computation, we set a threshold value for 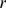, denoted by 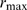, and reset the number of spikes as 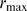 whenever a PN spikes more than this value. We set 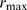 to 5 considering that the probability of there being more than five spikes within a bin is less than 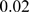.

The mutual information is given by
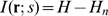
(7)

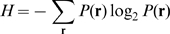
(8)

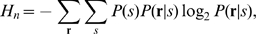
(9)where 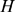 is the entropy and 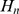 is the noise entropy and 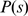 is uniform for all odors; that is, 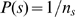, where 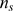 is the number of odors. We estimated the conditional probability distribution 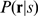 by simulating the network model 400 times. 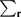 represents the summation over all possible PN activity patterns. The number of all possible PN activity patterns is 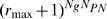, where 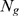 is the number of glomeruli and 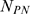 is the number of PNs within each glomerulus. The computational costs grow exponentially with the number of neurons, so the mutual information calculation is limited by the size of the neural population. When we computed the mutual information using Eqs. 7–9, we set 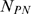 to 1 and 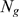 to 8.

When we considered a larger number of neurons (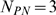, 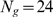), we estimated the mutual information by using the decoding approach [33]. In this approach, we trained support vector machine (SVM) classifiers and evaluated their performance. Decoding performance is usually quantified by the correct classification rate, which is the average of the diagonal elements in the confusion matrix. Whereas the classification rate deals with only the most likely stimulus predicted by the decoders given a particular neural response, the mutual information quantifies the overall knowledge about the presented stimulus, such as which stimulus is unlikely given a particular neural response. To link the information theoretic and decoding approaches, we must take into account the off-diagonal elements of the confusion matrix. We can estimate mutual information from the confusion matrix after decoding using the following equation [33], [34].
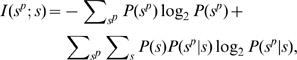
(10)where 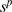 denotes the stimulus prediction of SVM classifiers for stimulus 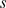 when the PN responses 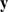 are given. Note that 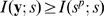 always holds from the data processing theorem [1]. Although the decoding approach underestimates the amount of information that neural responses carry, it can deal with much larger neural populations than methods that calculate the exact amount of mutual information. We used the information theoretic and decoding approaches in a complimentary manner to evaluate odor discriminability from neural responses.

A Library for Support Vector Machines (LIBSVM) was used to implement the SVM classifiers [35]. We used the one-against-one method for multiclass SVMs [36]. For 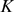 classes, this method constructs 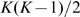 different 2-class SVM classifiers for all possible pairs of classes. Test points are then classified according to a majority vote of these 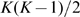 SVM classifiers as to which class is more likely. We chose a linear kernel because it gave the best classification performance and the closest estimate to the exact mutual information.

## Results

### Information theoretic approach

First, we computed the mutual information between odor stimuli and PN responses while systematically varying the intraglomerular transformation parameter 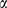 and LN input strength 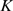 (see Eqs. 5 and 6). Although the actual average numbers of ORNs, 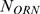, and PNs, 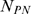, within a single glomerulus in the *Drosophila* antennal lobe are said to be 40 and 3, respectively [23]–[26], we set 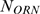 to 40 and 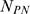 to 1 considering the cost of the mutual information computation. For the same reason, we reduced the number of glomeruli 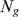 to 8 although data on ORN responses are available for 24 glomeruli (Fig. 2 (A)). We divided this data set into three non-overlapping groups consisting of eight glomeruli each and then computed the mutual information in these three groups. The number of LNs was set to 10. Later, we estimate the mutual information without reducing the number of neurons and using the entire data set at once (see ‘Decoding approach’).

**Figure 5:**
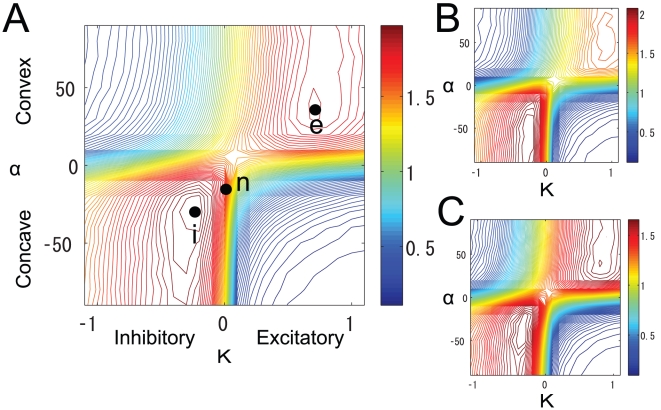
Contour plot of the mutual information when the intraglomerular transformation shape 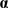 and LN input strength 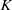 were varied. Colors show the value of the mutual information. Different sets of glomeruli were used in panels (A), (B), and (C). Peak e (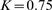, 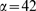), peak i (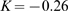, 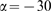) and point n (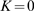, 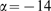) are the points where the mutual information was maximized under the conditions where 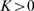, 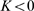, and 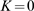 respectively.

Contour plots of the mutual information in a two-dimensional parameter space where the vertical axis is 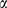 and the horizontal axis is 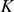 are shown in Figs. 5 (A)(B)(C). Although the plots are for computations on different sets of glomeruli, the results are qualitatively similar. Therefore, we focus on the results shown in Fig. 5 (A), where two peaks are prominent in this graphical representation of the mutual information. At the lower left peak (denoted peak i), 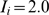, 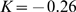, and 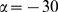, while at the upper right peak (denoted peak e), 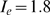, 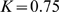, and 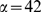. At peak i, the intraglomerular transformation is concave (dot-dashed line in Fig. 3) and the LN input is inhibitory. This combination of 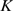 and 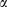 is consistent with previous experimental results for the *Drosophila* olfactory system [13], [31]. In contrast, at peak e, the intraglomerular transformation is convex (dashed line in Fig. 3) and the LN input is excitatory. There is less mutual information at peak e than at peak i, so the mutual information is maximized at peak i.

**Figure 6:**
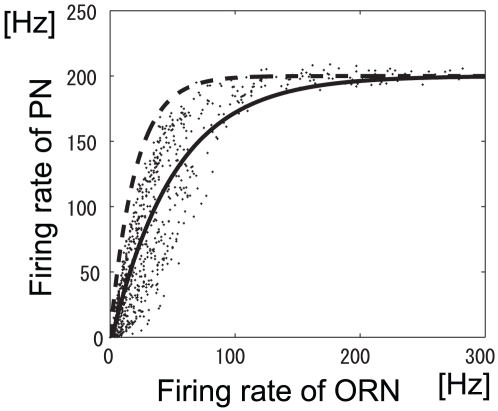
Relationship between ORN and PN responses at peak i. Dots show all types of ORN responses to all odors at peak i in Fig. 5 (A). The solid line is an exponential fit of the dots (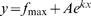). The dashed line shows the same relationship except with LN input set to 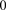 (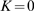).

The solid line in Fig. 6 shows the relationship between ORN and PN responses at peak i, and the dashed line shows the same relationship with the LN input removed. The nonlinear transformation shapes represented by the solid and dashed lines in Fig. 6 are similar to those observed in previous experiments [3], [13]. Olsen and Wilson [13] demonstrated the relationship between ORN and PN responses before and after removal of the lateral input, and these responses correspond to the solid line (before) and dashed line (after) in Fig. 6. This similarity in the nonlinear transformation suggests that from the viewpoint of information preservation, ORN activity is transformed in an almost optimal manner into PN activity in the *Drosophila* antennal lobe.

**Figure 7:**
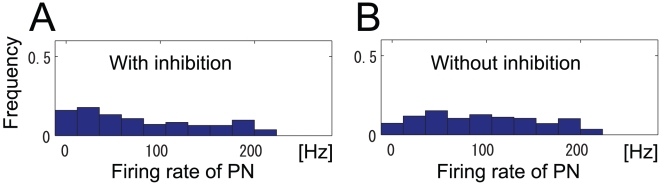
Histogram of PN responses. (A) Histogram of PN response magnitudes at peak i in Fig. 5 (A). (B) Histogram of PN response magnitudes at point n in Fig. 5 (A).

How the LN input affects the PN responses can be visualized by comparing the PN response histogram at peak i with that at point n, where mutual information is maximized under the condition of no LN input (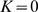) (Fig. 5 (A)). PN response histograms at peak i and point n are shown in Figs. 7 (A) and (B), respectively. As a consequence of the intraglomerular transformation, these histograms are flatter than the ORN response histogram (Fig. 2 (B)). However, by comparing these histograms, we can see that PN odor responses are slightly clustered around the weak end of the PNs' dynamic range at peak i. This is because only the intraglomerular transformation has an effect at point n, while the LN input has an additional effect at peak i. The PN response histogram shown in a previous experiment has similar characteristics to the histogram at peak i [3]. When mutual information at peak i is compared with that at point n, the value at peak i is larger than that at point n, 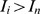 (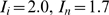). These results suggest that not only the intraglomerular transformation but also the LN input contribute to increase mutual information in the olfactory system as it did in our network model.

### Mechanisms underlying the enhancement of mutual information

Next, we examined how the intraglomerular transformation and the interglomerular interaction contribute to increase mutual information. Mutual information 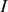 is the difference between entropy 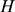 and noise entropy 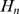 (Eq. 7). Entropy measures the variability of neural responses to different odors and is related to the degree of flatness in the histogram of the neural response magnitudes [3], [37]. Noise entropy measures the average variability of neural responses to a particular odor. For a large amount of mutual information to be obtained, entropy should be large and noise entropy should be small.

**Figure 8:**
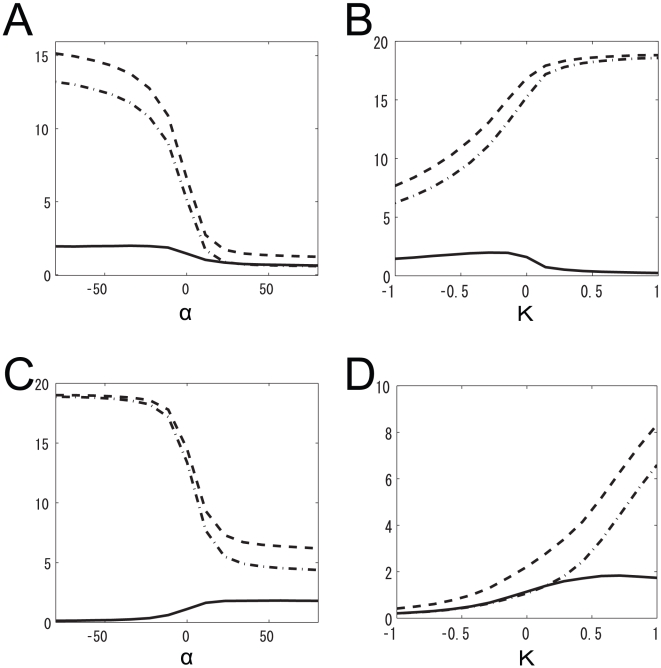
Dependence of the mutual information, entropy, and noise entropy on 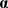 and 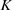. Solid lines represent the mutual information, dashed lines represent entropy, and dot-dashed lines show noise entropy. (A) 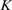 was set to the value at peak i in Fig. 5 (A) (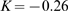). (B) 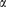 was set to the value at peak i in Fig. 5 (A) (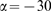). (C) 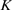 was set to the value at peak e in Fig. 5 (A) (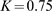). (D) 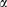 was set to the value at peak e in Fig. 5 (A) (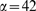).

We examined how mutual information, entropy, and noise entropy changed when 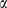 or 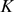 was changed around peak i in Fig. 5 (A). We found that both entropy and noise entropy increased as the intraglomerular transformation shape was changed from linear (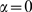) to concave (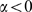) (Fig. 8 (A)). Mutual information increased because entropy increased more rapidly than noise entropy. This result indicates that the concave intraglomerular transformation increases mutual information by increasing the variability of neural responses to different odors. In contrast, both entropy and noise entropy decreased as the strength of inhibitory LN input increased (Fig. 8 (B)). Mutual information increased because noise entropy decreased more than entropy. This result indicates that the inhibitory LN input increases mutual information by decreasing the noise of neural responses.

When 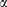 or 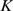 was changed around peak e, the behavior of the entropy and noise entropy was opposite to that around peak i. The convex intraglomerular transformation increased mutual information by decreasing noise entropy, and the excitatory LN input increased mutual information by increasing the entropy (Fig. 8 (C)(D)).

### Decoding approach

In the previous section, we used the subdivided data sets obtained from the data set in Fig. 2 (A). In this section, we describe the use of the whole data set containing ORN responses to 110 odors in 24 glomeruli. We also set the numbers of ORNs and PNs within a single glomerulus to 40 and 3, respectively, to match the actual average numbers of neurons in the *Drosophila* antennal lobe. The number of LNs was set to 10, as in the previous section. To assess a large number of neurons, we estimated mutual information using the decoding approach [33] rather than computing it exactly. To estimate mutual information in Eq. 10, we ran simulations of the olfactory network described in the previous section. We then trained linear SVM classifiers by using the simulation data set and tested their performance. Finally, we estimated mutual information from the performance of the linear SVM classifiers (see ‘Methods’ for details).

**Figure 9:**
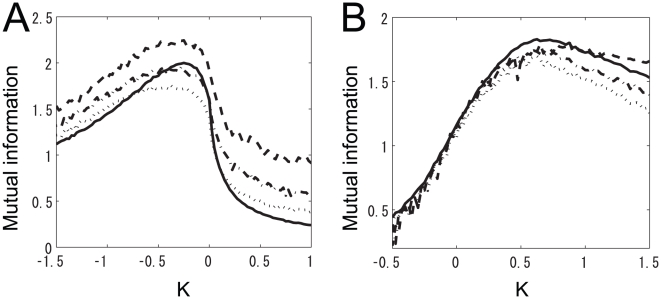
Comparison of actual and estimated mutual information. The solid line represents the actual mutual information. Dashed, dot-dashed, and dotted lines represent the estimated mutual information when the number of trainings and test data was 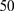, 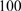, and 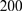, respectively. (A) 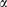 was set to the value at peak i in Fig. 5 (A) (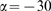). (B) 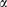 was set to the value at peak e in Fig. 5 (A) (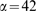).

First, we examined how well the mutual information estimated from the decoding approach matched the actual mutual information. We performed this comparison using the subdivided data set presented in Fig. 5 (A). Figures 9 (A) and (B) show the exact and estimated mutual information when 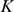 was changed around peaks i and e, respectively. Although the estimated mutual information converged to a level that underestimates the real mutual information, we were able to estimate with relatively high accuracy the positions of both peaks (Fig. 9). Therefore, with regard to the positions of and relationship between the peaks, the mutual information estimated from the SVM classifiers provides a reliable answer. We subsequently set the number of trainings and test data to 200 each. With this approach, we next estimated mutual information using the entire data set.

**Figure 10:**
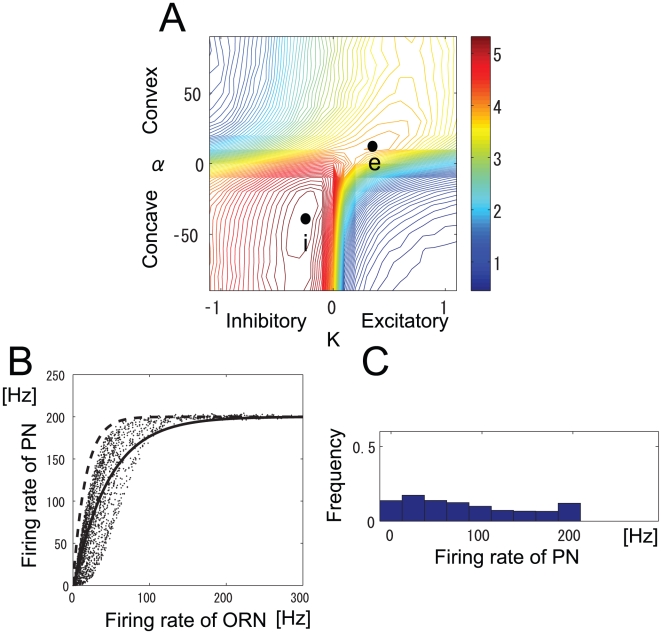
Results of the decoding approach: they are qualitatively the same as those of the information theoretic approach. (A) Contour plot of estimated mutual information when the intraglomerular transformation shape 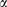 and LN input strength 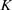 were varied. Colors show the value of the estimated mutual information. All glomeruli shown in Fig. 2 (A) were used. (B) Relationship between ORN and PN responses at peak i in panel (A). Dots represent all types of ORN responses to all odors. The solid line is an exponential fit of the dots (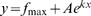). The dashed line represents the same relationship except with LN input set to 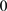 (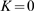). (C) Histogram of PN response magnitudes at peak i in panel (A).

A contour plot of the estimated mutual information is shown in Fig. 10 (A). As in Fig. 5 (A) there are two peaks. At peak i, 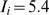, 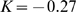, and 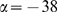; at peak e, 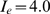, 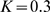, and 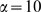. We increased the number of glomeruli, so these mutual information estimates are larger than those obtained in the previous section. There was significantly more mutual information at peak i than at peak e. Figures 10 (B) and (C) show the relationship between ORN and PN responses and the histogram of PN response magnitudes at peak i, respectively. The results in these figures qualitatively match the results obtained in previous physiological experiments [3], [13], further suggesting that the principle of maximum information preservation is used in the *Drosophila* antennal lobe.

To compare the coding efficiency in PNs with that in ORNs, we compared the mutual information of ORNs with that of PNs when the mutual information was maximized (at peak i). The estimated mutual information contained in all ORNs and in all PNs were 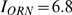 and 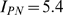; therefore, 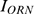 was larger than 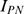, which is consistent with the data processing theorem [1]. When we computed the mutual information using the same numbers of ORNs and PNs, however, the estimated ORN mutual information became 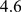, which is markedly smaller than 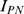. This demonstrates that PNs encode odor information more efficiently than ORNs at peak i. This is consistent with the experimental results of Bhandawat et al. [3].

### Adaptive gain control

As described in the ‘Methods’ section, the strength of the inhibitory lateral input is positively correlated with the total ORN activity evoked by each odor [13]. This lateral inhibition is considered to mediate gain control in the olfactory circuit. In this section, we discuss how the adaptive gain control promotes a more efficient neural code for odors by considering the discrimination of pairs of odors.

Table 1 shows how the inhibitory LN input changed the performance of binary SVM classifiers, the distance of mean responses, and the mean variance of responses for all possible pairs of odors. 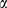 and 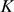 are values at peak i. The distance of the mean responses to two odors is the distance between two vectors of the mean number of spikes emitted by PNs within 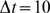 ms. The mean variance of PN responses to an odor is the mean of the distance between the trial-averaged PN response vector and the individual PN response vectors in all the trials. The number of trials was 1000. To enable the responses to different odors to be separated, the distance of mean responses should be large and the mean variance of responses should be low. As can be seen in Table 1, inhibition basically decreases the neural variability for all PN responses.

**Table 1:** Effects of inhibitory LN input on odor pair discrimination.

		Ratio(%)	Averaged firing rate without inhibition (Hz)	Averaged firing rate with inhibition (Hz)	Difference of averaged firing rate (Hz)	Difference of correct rate (%)	Difference of variance (Hz)	Difference of distance (Hz)
Correct rate increase	Distance increases and variance decreases	57	146	89	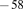	5.00	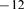	67
	Distance decreases and variance decreases	18	127	79	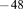	0.82	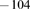	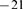
Correct rate decreases	Distance increases and variance decreases	2	130	80	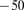	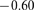	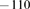	15
	Distance decreases and variance decreases	21	121	77	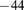	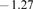	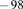	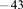
Correct rate does not change		2	123	78	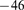	0	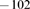	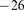

**Figure 11:**
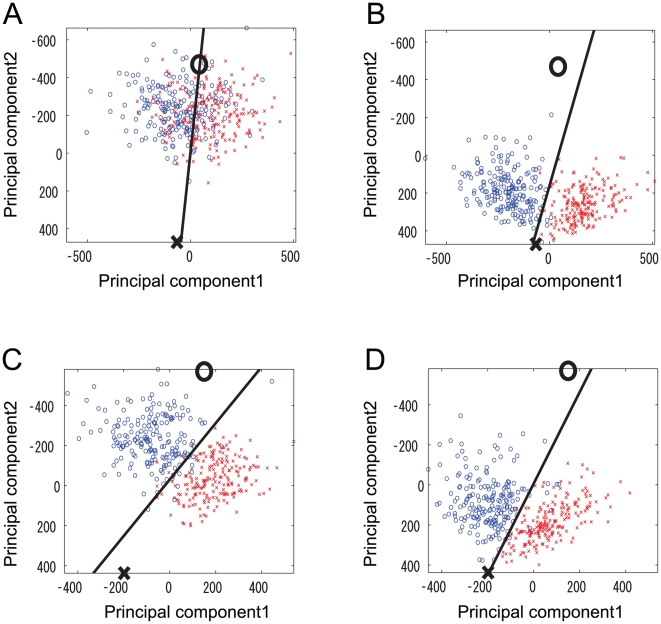
Visualization of the effects of an inhibitory LN input that separates one response from another. Two different inhibition mechanisms that distinguish odors as represented in panels (A)(B) and in panels (C)(D). Circles and crosses are simulated data for PN responses to two different odor pairs obtained from our network model. Responses of 24 PN types to the odors are projected onto a space defined by the first two principal components. Solid lines show the decision boundaries of SVM classifiers learned from training data. Test data are plotted. Large crosses at the bottom of the figures represent the points where all PNs were silent. Large circles at the top represent the points where all PNs were firing. 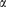 was set to the value at peak i in Fig. 10 (A) (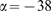). In panels (A) and (C), there was is no LN input (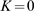). In panels (B) and (D), 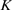 was set to the value at peak i in Fig. 10 (A) (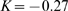).

We found that the correct classification rate was increased for 75% of pairs of odors by inhibitory LN input (Table 1). In 76% of cases within this category, the distance of mean responses was increased while the mean variance of responses was decreased, which are both beneficial for odor discrimination. These odor pairs evoked strong responses in ORNs. Since the inhibitory inputs were strong when the total ORN activity was high, these responses were strongly inhibiting. We visualized how the strong inhibitory LN input separated PN responses to odor pairs of this type by using principal component analysis. In Fig. 11 (A), where there is no inhibition (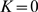), two clusters corresponding to the PN responses to two odor stimuli are concentrated near the point (large circle) where the firing rates of all PNs are maximum. This shows that many PNs received a strong input from ORNs when these two odors were presented. In this case, the distance between mean responses to two odors was small because of the saturation of responses caused by the concave intraglomerular transformation. When inhibition was induced, the two clusters separated and moved toward a point (large cross) where all PNs were silent (Fig. 11 (B)).

In the other pairs for which the correct classification rate was increased, the distance between mean responses was decreased. However, the correct classification rate was increased since the variability of neural responses was also decreased. The PN responses of pairs of these types are shown in Figs. 11 (C)(D). In these pairs, PN firing rates were relatively low, which means that the inhibition was not strong. In Fig. 11 (D), the distance between the center points of clusters is decreased as well as variability of neural responses compared with Fig. 11 (C). However, the amount of the increase in the correct classification rate is relatively small.

In 21% of pairs, the correct classification rate was decreased due to the decrease in the distance between mean responses. For these pairs, the inhibitory input was small because PN firing rates were relatively low. The amount of the decrease in the correct classification was also relatively small. In 2% of the odors, the correct classification rate did not change. In these pairs, the correct classification was 100% with or without inhibition.

Taken together, these results indicate that an inhibitory LN input enhances odor discriminability mainly by separating the responses of PNs that receive a strong ORN input. Without lateral input, these PN responses saturated because of the concave intraglomerular transformation. For odors where the total ORN activity was relatively small, inhibitory LN input did not affect odor separability much because the amount of inhibition was not high. In this case, the separability of odors was increased for some of pairs (18%) and decreased for some of pairs (21%). On the whole, adaptively changing the inhibitory LN input helps odor discrimination.

### Effect of static firing threshold on mutual information

**Figure 12:**
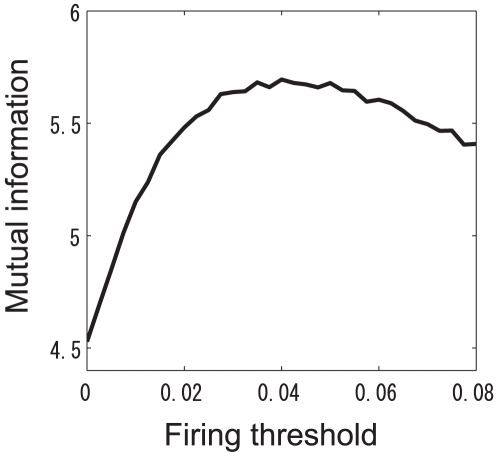
Dependence of the mutual information on the PN firing threshold. The intraglomerular transformation shape 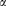 was fixed at 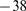.

In the previous sections, we assumed that the PN firing threshold 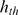 was fixed at 0, reflecting the experimental observation [13] that the slope of a firing rate transformation curve was very steep even when the ORN firing rate was close to 0. In this section, we report on varying 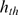 and investigating the effects of raising the firing threshold. First, we examined how increasing 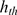 affected the mutual information when 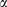 was fixed. The mutual information was estimated by using the decoding approach, as in the previous section. The estimated mutual information when 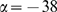 is shown in Fig. 12. The mutual information was maximized when 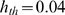. Thus, raising the PN firing threshold can increase the mutual information like increasing the strength of adaptive inhibitory inputs can.

**Figure 13:**
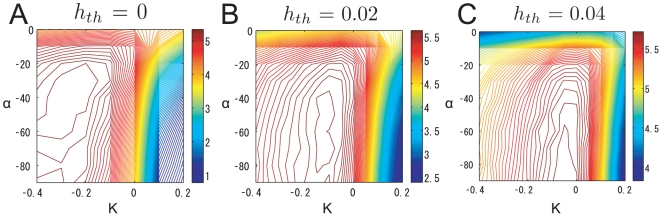
Contour plot of estimated mutual information when the firing threshold was changed. The intraglomerular transformation shape 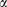 and LN input strength 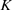 were varied. (A) 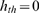. (B) 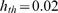. (C) 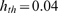.

The contour plot of the estimated mutual information when 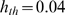 is shown in Fig. 13(C). In this case, the mutual information was maximized when 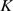 was nearly 0, and the beneficial effect of LN input on the mutual information was significantly diminished. This is because the PN firing rates were already fairly suppressed by the firing threshold. When 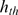 was smaller than the optimized value (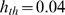), the mutual information was maximized in a region where inhibitory gain control worked. For instance, when 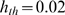 (Fig. 13(B)), we can see an i peak, as in the case of 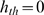 (Fig. 13(A)).

The firing transformation between ORNs and PNs when 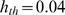 and 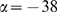 is shown in Fig. 4. There, the slope at the origin is nearly 0, which is inconsistent with the experimental data [13]. We therefore could conclude that the PN firing threshold in the actual olfactory system is smaller than this optimized value and that an adaptive inhibitory input can promote efficient neural coding of odors (see ‘Discussion’).

## Discussion

### Maximum information preservation in the *Drosophila* antennal lobe

In this study, we investigated whether information related to odor stimuli is maximally preserved in the *Drosophila* antennal lobe. Taking account of approximately half of all the neurons engaged in olfactory processing (24 out of a total of approx. 50 glomeruli) and ORN responses to 110 odorants, we computed the mutual information between odor stimuli and PN responses in an antennal lobe model. Our network model is simple but incorporates the essential architecture and connectivity of the antennal lobe. We found that mutual information was maximized when the intraglomerular transformation was concave (Fig. 3) and the LN input was inhibitory, which is consistent with previous experimental results [13], [31]. Furthermore, the shape of the resultant nonlinear transformation between ORN and PN responses is similar to that observed experimentally [3], [13]. This indicates that the principle of maximum information preservation is used in the *Drosophila* primary olfactory center.

### Neural mechanisms underlying maximum information preservation

We also examined how the intraglomerular transformation and inhibitory LN input contribute to increase the mutual information. In ORNs, odor responses are clustered at the weak end of their dynamic range. The concave intraglomerular transformation increases mutual information by equally distributing PN response magnitudes in their dynamic range. In terms of entropy and noise entropy, the concave intraglomerular transformation increases mutual information by increasing entropy more than noise entropy (Fig. 8 (A)).

Inhibitory LN input has two beneficial effects. The first is to decrease the neural variability of PN responses evoked by a given odor, as shown in Table 1. The second is to separate saturated PN responses by inhibiting them (Figs. 11 (A)(B)). Importantly, the inhibitory LN input is adaptive, i.e., the inhibitory input strength depends on the overall ORN activity [13]. This adaptive gain control mechanism enables the actual olfactory system to deal with odors with a wide range of magnitudes. Raising the PN firing threshold, which can be considered as static inhibition, can increase the mutual information like adaptive inhibitory LN input can (Fig. 12). However, raising the firing thresholds has the disadvantage that it equally inhibits PN responses regardless of the magnitude of ORN responses whereas adaptive inhibition does not inhibit weak PN responses much when the total ORN activity is low. This will prevent the brain from recognizing low-concentration odors. Because the olfactory system has to deal with a wide variety of odors, we infer that the firing threshold of a real PN is low and that adaptive gain control mechanisms, rather than a static threshold, are used. In fact, we found that the firing rate transformation between ORNs and PNs when the PN firing threshold was high did not resemble the actually observed one (Fig. 4).

### Two possible mechanisms promoting odor discrimination

We computed the mutual information between stimuli and PN responses by systematically changing the parameters of intraglomerular transformation and LN input strength. We found two peaks in the graphical representation of the mutual information (Fig. 5 (A)). At one of them (peak i), the intraglomerular transformation is concave (dot-dashed line in Fig. 3) and LN input is inhibitory, which is consistent with the experimental results. At the other (peak e), the intraglomerular transformation is convex (dashed line in Fig. 3) and the LN input is excitatory. Although both of these neural mechanisms promote odor discrimination, the combination at peak i is used in the *Drosophila* olfactory circuit. One reason for the use of this combination is demonstrated by our finding that the peak value of mutual information at peak i is higher than that at peak e. Another reason is that excitatory LNs cannot perform adaptive gain control. If the net LN input is excitatory, the olfactory system cannot discriminate between odors over a wide range of concentrations or odor mixtures. For these reasons, the combination of concave intraglomerular transformation and inhibitory LN input can be considered the most appropriate in the olfactory circuit.

### Robustness against change in nonlinear firing rate transformation shape

In Fig. 8 (A), which shows the dependence of mutual information on the intraglomerular transformation shape, we can see two significant features. One is that the mutual information decreases rapidly as parameter 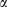 increases and approaches the region where the transformation function is convex (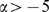). The other is that the mutual information changes little in the wide region where the transformation function is concave (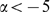). These features indicate that the *Drosophila* olfactory system is robust against changes in the shape of the intraglomerular firing transformation for odor discrimination provided that the transformation is concave.

Bhandawat et al. [3] examined the shapes of the nonlinear transformation between ORN and PN firing rates in seven different glomeruli and observed two features similar to those observed in our network model. First, the shape was concave in every glomerulus. Second, these shapes showed some degree of variation. From the viewpoint of odor discrimination, our results provide explanations as to why the shape of the nonlinear transformation between ORN and PN responses should be concave in every glomerulus and why the nonlinear transformation shapes could differ from glomerulus to glomerulus as long as they are concave.

### Approaches for understanding neural mechanisms

We demonstrated that the optimum nonlinear firing rate transformation between ORNs and PNs obtained by maximizing mutual information is similar to that observed in previous experiments (Figs. 6 and 10 (B)). Similarly, in many previous studies, it has been reported that optimum neural representations of sensory stimuli, which are predicted theoretically, resemble the actual response properties of early sensory neurons [38]–[42]. In this study, however, we investigated not only the optimum information transmission from the viewpoint of information maximization, but also the mechanisms of information maximization in the neural circuit, which had not previously been rigorously theoretically investigated.

We studied them by taking a different approach from previous studies to obtain optimum information transmission. First, we used actual physiological data as input stimuli. Second, we constructed an experimentally grounded network model of the *Drosophila* olfactory circuit and computed the mutual information between stimuli and PN responses in that network model. Third, by systematically changing the network's parameters, we searched for the neural mechanism that maximized the mutual information. This approach was possible owing to the characteristic advantages in the *Drosophila* olfactory circuit, namely a simple glomerular structure, a relatively small number of neurons engaged in sensory processing, and well studied response properties and connectivity of those neurons. By using this approach, we showed that the neural mechanisms underlying information maximization are consistent with previous experimental results. That is, when mutual information is maximized in the network model, the shape of the intraglomerular function is concave and the net LN input is inhibitory.

For the sake of simplicity, we used a simple neuron model and did not implement realistic LN inputs [12]–[16] or synaptic depression and refractory periods, which are thought to be the main origins of the concave firing rate transformation within glomeruli [3], [31]. In the future, realistic implementation of synaptic depression and LN interactions should give us a more detailed understanding of the nature of maximum information preservation in actual biological systems. This should also enable us to compare theoretical and experimental results in a more quantitative manner. It will be interesting to further investigate how maximum information preservation is implemented in the olfactory circuit in light of the basic findings obtained from this study.
